# Methodological Approach to Improve Surgical Outcomes of a Pig Subretinal Implantation Model

**DOI:** 10.1167/tvst.11.4.24

**Published:** 2022-04-29

**Authors:** Fukutaro Mano, Jarel K. Gandhi, Raphael Pereira da Silva, Aline Do Amaral Silva, Lucas Iezzi, Raymond Iezzi, Jose S. Pulido, Alan D. Marmorstein

**Affiliations:** 1 Department of Ophthalmology, Mayo Clinic, 200 First Street SW, Rochester, MN, USA

**Keywords:** cell therapy, pig, surgery

## Abstract

**Purpose:**

To improve outcomes for subretinal implantation surgery in pigs.

**Methods:**

Analysis of variables affecting the success of subretinal implantation surgery was performed on videos of 37 surgeries. Ex vivo experiments were conducted to measure intraocular pressure (IOP) and test various prototyped implanters for effectiveness at maintaining IOP.

**Results:**

A video analysis revealed a prolonged sclerotomy open time owing to a combination of uncontrolled bleeding and excessive fluid outflow often resulting in retinal prolapse. Precauterization of the choroid before full-thickness sclerotomy (*n* = 10) resulted in a reduced incidence of uncontrolled bleeding from 39.1% (9/23) versus 0% (0/10) (*P* = 0.005) and improved implantation success from 73% to 90%. An ex vivo analysis of the IOP revealed a mean decrease in the IOP from 30.2 ± 3.0 mm Hg to 5.0 ± 2.1 mm Hg after a fully penetrating sclerotomy. To address this situation, we produced a series of plugs that integrated with a custom implant insertion device to seal the sclerotomy during implantation. The use of the plugs was cumbersome, however, and so we opted instead to increase the width of the inserter tip to fill the open sclerotomy. This improved device restored and maintained IOP during implantation (27.1 ± 1.9 mm Hg). Combined with precauterization the improved inserter resulted in 100% successful implantation (*n* = 4).

**Conclusions:**

For subretinal implantation in pigs, a modified procedure to precauterize the choroid before sclerotomy combined with an instrument that better fills the scleral opening decreases bleeding, hypotony, and open sclerotomy time, improving the success rate.

**Translational Relevance:**

Better management of IOP and bleeding from a sclerotomy will improve implant-based therapies.

## Introduction

Retinal pigment epithelium (RPE) dysfunction is considered a critical factor in the pathogenesis of age-related macular degeneration (AMD).^1^ In recent years, research has focused on cell-based treatments for AMD to replace this vital support.^2^^–^^5^ Three recent phase I human trials have demonstrated the safety of the transplantation of stem cell-derived RPE, although the efficacy remains to be investigated thoroughly.^3^^–^^5^ Similarly, the surgical translocation of autologous RPE–choroid tissue from a midperipheral lesion to the macula has been reported, with one case achieving a visual acuity of 20/30 even 13 years after the translocation.^6^ However, autologous RPE–choroid tissue translocation surgery is considered to be highly challenging as it requires a more than 180° retinotomy and is often followed by high rates of postoperative complications. Nevertheless, both stem cell–derived RPE transplants and RPE–choroid translocation have shown potential for treatments of AMD. In developing cell-based treatments for AMD, the use of the pig model has gained considerable traction. For example, several groups have recently used pigs to investigate instrument development and generate proof of concept or safety data.^7^^–^^9^ Other investigators have also investigated or produced pig models of geographic atrophy.^10^^,^^11^ Domestic pig eyes have an anatomy that is similar to the human eye, allowing for the use of human surgical instruments and the standard vitrectomy setup. Pigs also have an area centralis that is low on vasculature and suitable for simulated macula-like implantation.

We recently reported the use of a pig model to perform the subretinal implantation of fibrin hydrogels that degraded spontaneously within 8 weeks of placement in vivo, the first report to show a full-time course of degradation of a scaffold in the subretinal space without any adverse effects to the retinal environment.^12^ While performing this work, we encountered many of the challenges in adapting to a pig model of subretinal surgery. In establishing a standardized surgical method, we initially encountered significant complications, such as unplanned retinal detachments and subretinal or choroidal hemorrhages.

We hypothesized that these adverse outcomes were due to the prolonged time of scleral incision opening. We observed that the prolonged time of sclerotomy opening was caused by the need to address bleeding occurring from the underlying choroid and resulted in instability of intraocular pressure (IOP), which caused hypotony, bleeding from the retinotomy site, and/or retinal detachment. Thus, we set out to investigate methods to reduce these complications. We first addressed the bleeding at the sclerotomy site by modifying our surgical procedure to include a scleral dissection, precauterization of the underlying choroid, preplaced sutures, and finally a full-thickness sclerotomy. This method significantly shortened the sclerotomy open time and the incidence of adverse effects. However, it did not eliminate retinal detachments completely. As such, we performed ex vivo experiments to understand the fluctuation and instability of the IOP during the procedure and generated prototype surgical devices to improve IOP stability with the goal of diminishing or eliminating retinal detachment as a complication of subretinal implantation surgery. The resulting modified surgical procedure and device were used to successfully implant fibrin–scaffolded induced pluripotent stem cell (iPSC)–RPE without any adverse effects.

## Methods

### First-Generation Subretinal Implantation Device

**Figure 1.**
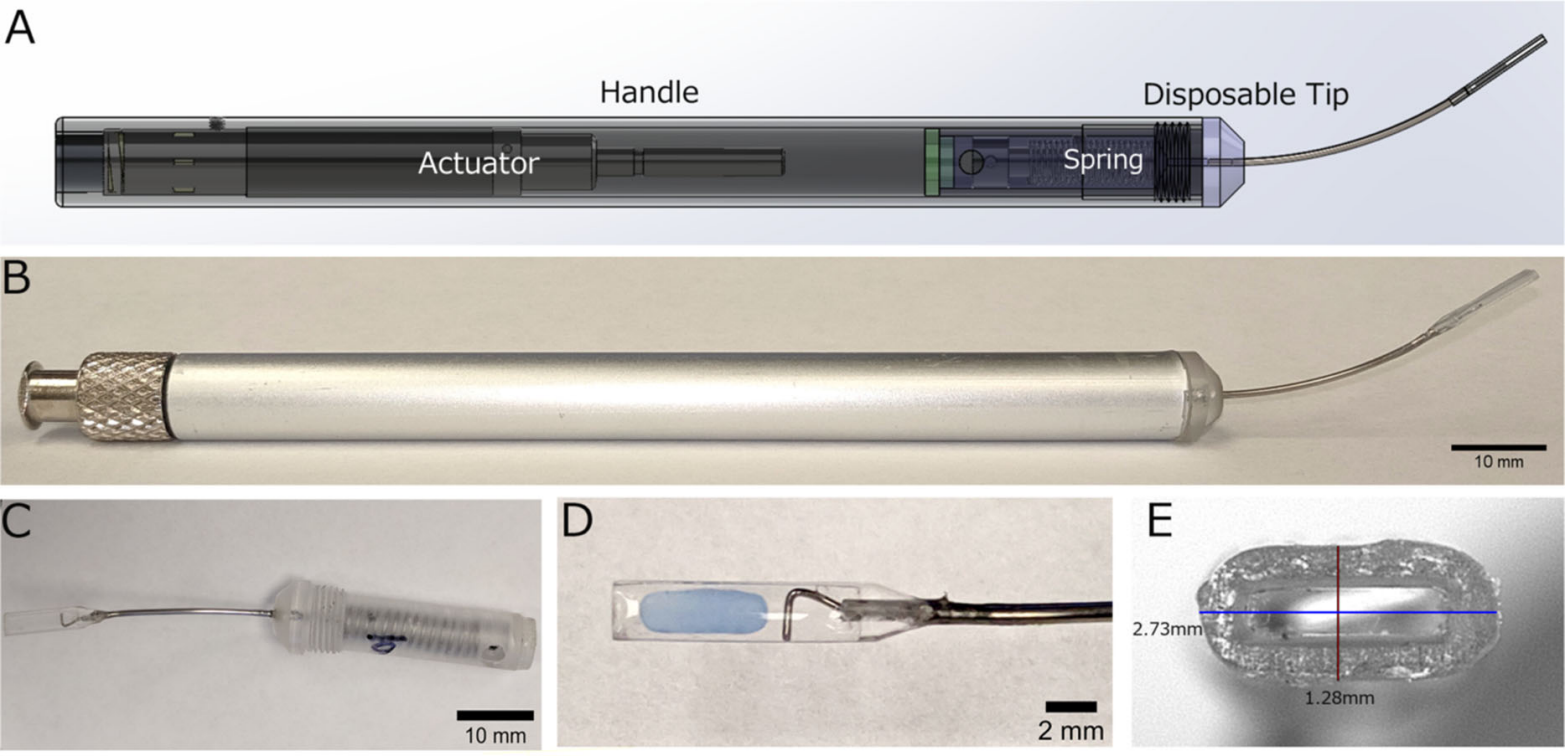
First generation subretinal implantation device. (A) CAD rendering of implantation device handle and installed disposable tip. Rendering is see-through to visualize the inner working parts. (B) Photograph of actual implantation device handle with an installed disposable tip. (C) Photograph of disposable tip consisting of a plastic hub (*right*) containing a spring and pin assembly. (D) Photograph of the clear plastic tip housing with a preloaded fibrin gel (*blue*). The stainless steel wire plunger is visible to the right of the gel. (E) Photograph showing the cross-sectional opening of the plastic tip housing with labeled dimensions.

A previously described implantation device was used for the initial experiments.^12^ This device consists of two parts: a reusable handle and a disposable surgical tip (Figs. 1A, B). Both parts were prototyped with assistance from Elite Custom Solutions (Rochester, MN).

The reusable handle contains a pneumatic actuator that is driven by fluid pressure via Luer Lock tubing attached to a syringe or viscous fluid control system (Alcon, Fort Worth, TX) and can be actuated using a third party manually or by foot pedal per the surgeons’ preference. The actuator is connected to a pin that docks to the disposable tip to push the plunger forward.

The disposable tip assembly (first generation) consists of a plastic hub containing a spring and pin assembly for the plunger (Fig. 1C). The plunger, a stainless steel wire, is thread through a stainless steel hub (23 G) and into a clear plastic tip housing. The clear plastic tip housing was 3D printed using a soft lithography technique with WaterShed XC 11122 (Somos; Elgin, IL). The implant is a 1.5-mm wide by 5.0-mm long by 200-µm thick fibrin hydrogel (Fig. 1D). To create a housing for the implant with sufficient clearance to load without scraping or sticking to the inner surface, we decided on an inner cross-section of 400 µm tall × 1.8 mm wide. The thickness of the walls is approximately 400 to 500 µm. The outer dimensions are 1.28 mm tall by 2.73 mm wide (Fig. 1E).

Fibrin hydrogel implants were formed as described previously.^12^ A custom punch was used to generate 1.5 mm × 5.0 mm geometry. To load the device, the clear plastic tip housing is filled with balanced saline solution (BSS) and the fibrin implant is loaded manually using forceps.

### General Surgical Procedures

Female domestic pigs (20–30 kg) were used to establish the surgical model as previously described.^12^ Before surgery, the pigs were screened under anesthesia for cataract, cupped optic disk and/or glaucoma, and iris neovascularization. All animal procedures were performed in accordance with the guidelines of the Association for Research in Vision and Ophthalmology Statement for the Use of Animals in Ophthalmic and Vision Research and received prior approval from the Mayo Clinic Animal Care and Use Committee (IACUC).

**Figure 2.**
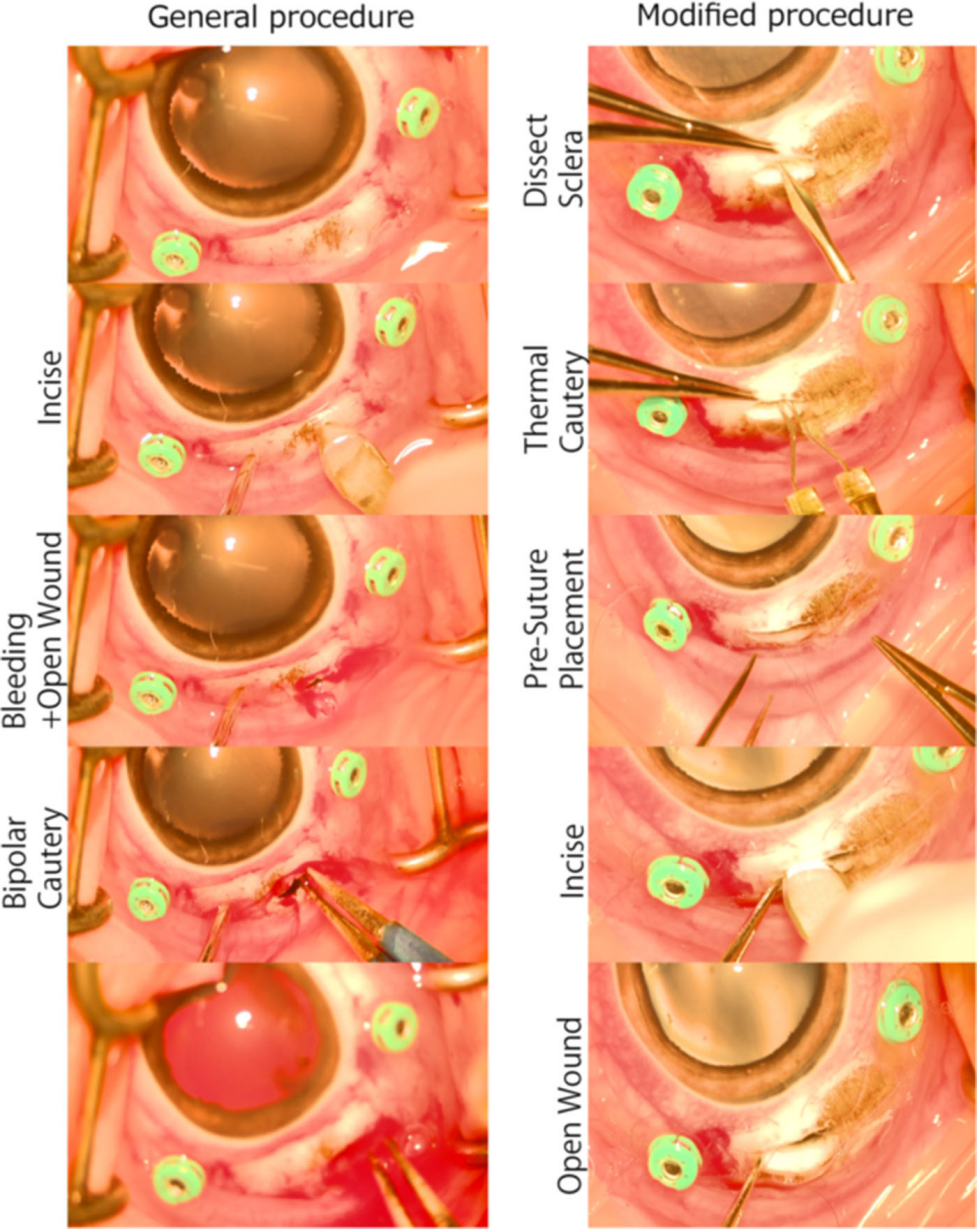
Schematic of scleral incision within both surgical procedures. The general procedure (*left column*) consists of a full scleral and choroidal incision using a slit knife, followed by bipolar cauterization to stop bleeding within the open wound. The modified procedure (*right column*) consists of dissecting the sclera, applying thermal cautery to the underlying choroid, the placement of presutures, and a full-thickness incision of the choroid with the slit knife. Application of the thermal cautery reduces the risk of choroidal bleeding after the incision (*bottom right*).

A subretinal implantation was performed as previously described^12^ and is referred to as the standard technique (Fig. 2). Pigs were submitted to general anesthesia using initial intramuscular xylazine (2 mg/kg) and telazol (5 mg/kg). Inhalant isoflurane (1.0%–3.5%) was used to maintain sedation throughout the surgery. Buprenorphine (0.18 mg/kg) and rimadyl (4 mg/kg) was provided intramuscular before surgery onset. The right eye was dilated using 1% tropicamide and 2.5% phenylephrine eye drops. A lateral canthotomy was performed temporally to facilitate surgical manipulation. A standard three-port vitrectomy setup was placed at 3.5 mm far from the limbus, with a fourth port for chandelier illumination (Alcon). A 25-G infusion cannula was used. A lens-sparing vitrectomy was performed. Staining of the posterior hyaloid was performed using up to 0.5 mL triamcinolone acetonide (Triesence 40 mg/mL; Alcon) followed by a posterior hyaloid detachment until no residual vitreous was confirmed on the retinal surface. After the partial vitrectomy, a retinal bleb was created using a 38 G or 40 G soft tip cannula (MedOne; Sarasota, FL) to inject BSS using the viscous fluid control system. Endodiathermy was performed at the edge of the bleb followed by a 1.5 mm retinotomy using 25 G vertical scissors (BVI Medical, Waltham, MA).

A nasal 3.6-mm width sclerotomy was performed using a slit knife (Alcon). An external cautery was applied to the incised choroid to control bleeding as necessary. The preloaded surgical tip was inserted through the sclerotomy, aligned to the retinotomy, and the actuator was engaged to deploy the implant into the subretinal space. After hydrogel placement, the sclerotomy was sutured closed. ILM forceps were used to adjust the location of the implanted gel if needed. In some cases, perfluorocarbon liquid (PFO) was used to flatten the retina over the implant. After a fluid–air exchange, the eye was filled with 20% sulfur hexafluoride (SF_6_) or silicone oil (Alcon).

### Surgical Outcomes Measurement

Raw surgery videos were reviewed by a qualified ophthalmologist to assess the incidence of complications. Outcomes measured included retinal detachment, bleeding from either retinotomy site or sclerotomy site, successful implantation, and total time of transplant defined as time from incision to wound closure.

### Modified Surgical Technique

Based on an analysis of adverse effects, the surgical approach was modified to minimize the open wound time by altering the sclerotomy technique (Fig. 2). The modified procedure expanded the sclerotomy step by first performing a partial thickness scleral dissection and then applying low-temperature cautery (Bovie) to the underlying choroid. Followed cauterization a full thickness incision was made using a slit knife (Alcon). All other steps were performed as described above.

Initially, 23 cases of transplantation surgery were performed with standard surgical procedures. A subset of this group included 16 cases that were published previously as a part of a study demonstrating that fibrin hydrogels will degrade spontaneously within 8 weeks of surgical implantation.^12^ Subsequently, 10 cases received fibrin transplantation with modified surgical technique and another 4 cases received iPSC–RPE cells seeded on the fibrin scaffold with modified surgical technique for the nonsurvival protocol. Because the iPSC–RPE cells accelerate the fibrin degradation, the subretinal implantation of much thinner iPSC–RPE graft was technically more difficult than that of just the fibrin sheet. Further, the four cases were all nonsurvival cases and excluded for the comparison of surgical outcomes between the standard and the modified procedures.

### IOP Stabilizer Plugs Generation

Two different devices were generated as a means to plug the sclerotomy site in attempts to maintain the IOP during the implantation. These two devices serve as snap-on attachments that dock with the stainless steel hub of the inserter tip (Supplementary Fig. S1). Both devices were designed to be free floating so that they can initially dock with the sclerotomy but permit the inserter tip to slide further into the eye as needed to line up with the retinotomy.

Two different designs were generated as a three-dimensional model using Solidworks (SolidWorks Corp, Waltham MA). The Type A plug (referred to as the PLA plug; Supplementary Fig. S1) was printed with polylactic acid (PLA) filament (MatterHackers, Lake Forest, CA) using a Prusa i3 MK3 3D printer (Prusa Research, Prague, Czech Republic). A scalpel was used to clean the edges of the device to generate a smoother surface. The second design was used to generate a negative mold for a Type B plug (referred to as the PDMS plug; Supplementary Fig. S1). The negative mold was printed with poly-ISO filament using a Poly-Jet printer (Stratasys, Rehovet, Israel). To generate the PDMS plug, silicone elastomer (Sylgard 184, Dow Corning, Midland, MI) was prepared per the manufacturer's protocol and degassed under vacuum for 30 minutes. A silicone-releasing agent (smooth-on) was sprayed within the negative mold. The negative mold was then filled with the silicone elastomer and a 23 G needle was inserted through the center of the mold to allow the plug to snap onto the inserter shaft. The Type B plug was then cured at 100°C for 2 hours. Once cooled, the plug was removed from the mold, and a slit was cut lengthwise on the side to allow the plug to slip onto the inserter tip shaft.

### Ex Vivo Experiment for IOP Stabilization During the Gel Deployment

IOP measurement experiments were performed with 10 cadaver pig eyes. Freshly enucleated pig eyes were provided by the Hormel Institute (Austin, MN), and the experiment was scheduled within 6 hours from tissue procurement. The cadaver eyes were pinned down for surgical manipulation. To enable visualization, a pars plana lensectomy was performed. A vitrectomy was then performed as described elsewhere in this article. Real-time IOP measurements were performed using a modification of our prior protocol for mice.^13^ To accomplish this, the posterior chamber was cannulated with a 25 G needle (Becton Dickinson, Franklin Lakes, NJ) placed through the pars plana with the tip at the center of the globe. The tip of the needle was visually confirmed as beveled up before recording measurements. The needle was connected to a DTXplus pressure transducer (Merit Medical, Singapore) and calibrated to a water height equivalent of 0 mm Hg. The transducer signal was converted and recorded using a custom DAC converter hardware and software created by the Mayo Clinic Division of Engineering.^14^ The IOP measurements were calibrated using the Accurus gravity pole setup and confirmed before each experiment.

The IOP measurements were recorded at key events during the procedure: background IOP before sclerotomy, after the scleral incision made using a slit knife (incision), insertion of the clear rectangular tip of the inserter (tip in), full insertion of the device to line up with the retinotomy (all in), and complete removal of the insertion (Supplementary Fig. S2). This procedure was performed without an IOP stabilizer, and with the PLA plug and the PDMS plug. A final IOP measurement was taken after a single 8-0 Vicryl suture was placed in the middle of the scleral incision. It was necessary to perform a vitrectomy around the sclerotomy site after the initial incision to ensure more consistent IOP recordings. The average IOP was calculated from a consecutive 10-second recording during each phase. Compliance of the setup, including the pig eye, was assumed to fall within the standard deviation of each group.

### Updated Surgical Tip

**Figure 3.**
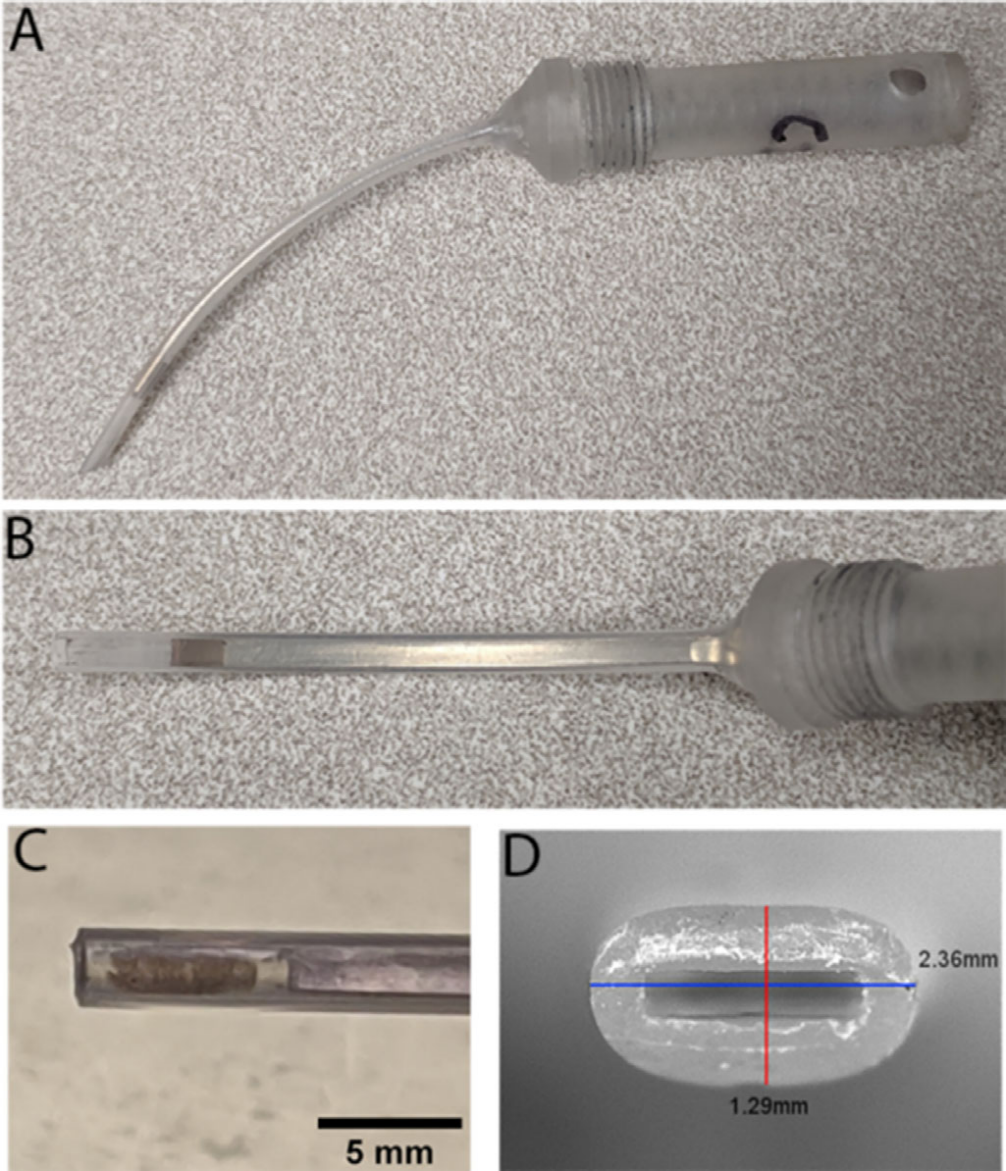
Second-generation surgical tip. (A) A second-generation surgical tip compliant with the previous device handle was developed to maintain the IOP during deployment. (B) The hub of the disposable tip was designed to cover the same outer dimension for the full length of the device. (C) Photograph of distal end showing an MRPE611 implant consisting of iPSC-RPE monolayer on a fibrin gel. (D) Photograph showing the cross-sectional opening of the nylon body with labeled dimensions.

Based on the ex vivo IOP data, a newer iteration of the surgical tip was prototyped (Fig. 3). The disposable tip part was designed to be compatible with the reusable handles described above. The new surgical tip device (second generation) was prototyped with assistance from Meddux Development Corp (Boulder, CO). The key element modified was to use one nylon extrusion to seamlessly combine the implant housing and shaft/hub. This single piece maintains the same outer dimension for the full length of the device. The previous stainless steel wire plunger was replaced with a flat stainless steel metal strip. The PDMS seal was replaced with Teflon tape seal at the end of the plunger. A total of two batches were produced (batch P and T), with three prototypes in each batch, with variations on the bevel angle (0°–45°) or hub curvature radius (25–45 mm). The ex vivo IOP measurements were repeated with each prototype using the protocol described elsewhere in this article.

### Manufacture of MRPE611

The iPSC-RPE cells were obtained from LAgen Laboratories (Rochester, MN) and cultured on top of a fibrin hydrogel as previously described.^15^ Cells were cultured for a minimum of 30 days in RPEM media (LAgen Laboratories) supplemented with 2.5 mg/mL tranexamic acid (Sigma-Aldrich, St Louis, MO), B27 supplement (ThermoFisher Scientific, Waltham, MA) and antibiotic–antimycotic (ThermoFisher Scientific). For use, implants were aseptically processed to create 1.5 × 5.0-mm implants, loaded into the newer surgical tips before use.

Implantation of MRPE611 was performed in live domestic pigs as described above using the modified surgery technique and a second-generation implantation tip (*n* = 4). After implantation, perfluorocarbon liquid was used to flatten the retina above the implant to visualize the implant. Owing to the xenogeneic nature of implanting human cells into a pig model, the animals were sacrificed immediately at the conclusion of the surgery as a nonsurvival procedure.

### Statistics

Data are reported as mean ± standard deviation. Statistical analysis was performed using JMP 14 software (JMP; SAS, Cary, NC). The comparison of the surgical complications between groups was assessed by the χ^2^ test. A one-way analysis of variance was used to compare the total time of transplant. For all experiments, significance was established with a *P* value of less than 0.05.

## Results

### Surgical Outcomes of Fibrin Hydrogel Implantation

Table 1 highlights the surgical outcomes. Overall, of 23 cases, 11 reported unintended retinal detachment (48%), 9 reported bleeding from the retinotomy site (39%), and 5 reported uncontrollable bleeding from the sclerotomy site by external cautery (22%). Of 23 cases, 17 resulted in successful implantation (74%), with 13 cases resulting in survival (57%). After initially detecting choroidal bleeding during the scleral incision, an experiment was conducted to determine the location of ora serrata from the limbus in each quadrant in cadaver pig eyes. Although on average, the ora serrata was 3.5 mm from the limbus, the variability was far too great to be predictably accurate during live surgery. We did not observe any direct association with animal size or age.

**Table 1.** Surgical Outcomes of Fibrin Hydrogel Transplantation in Each Case

Animals	Use of Choroidal Cautery	Intraoperative RD	Bleeding From Retinotomy	Bleeding From Sclerotomy	Successful Implantation	Tamponade Materials Used	Eye Opening Time (s)	Survival	Postoperative RD
1	No	Yes	No	No	Yes	None	494	Yes	Yes
2	No	No	No	No	Yes	None	539	Yes	Yes
3	No	Yes	Yes	No	No	Air	691	No	
4	No	No	Yes	Yes	Yes	None	253	No	
5	No	No	Yes	No	Yes	None	304	No	
6	No	No	No	No	Yes	None	793	Yes	Yes
7	No	Yes	Yes	No	No	Air	465	No	
8	No	No	No	No	Yes	None	431	Yes	No
9	No	Yes	No	No	No	None		No	
10	No	No	No	No	Yes	None	674	Yes	No
11	No	No	No	No	Yes	Air	754	Yes	No
12	No	Yes	No	No	Yes	Air	265	Yes	Yes
13	No	Yes	No	No	Yes	Air	1029	Yes	Yes
14	No	No	No	No	Yes	SO	461	Yes	Yes
15	No	No	No	No	Yes	SO	425	Yes	No
16	No	No	No	No	Yes	SO	1501	Yes	No
17	No	No	Yes	Yes	Yes	None	219	No	
18	No	Yes	Yes	No	Yes	None	505	No	
19	No	Yes	Yes	Yes	No	Air	1026	No	
20	No	Yes	Yes	Yes	No	None		No	
21	No	No	No	No	Yes	Air	298	Yes	No
22	No	Yes	Yes	No	No	None		No	
23	No	Yes	No	Yes	Yes	Air	250	Yes	Yes
24	Yes	No	No	No	Yes	Air	200	Yes	No
25	Yes	No	No	No	Yes	PFO/air	125	Yes	No
26	Yes	No	No	No	Yes	Air	116	Yes	No
27	Yes	No	No	No	Yes	PFO/air	167	Yes	No
28	Yes	Yes	No	Yes	No	None		No	
29	Yes	No	No	No	Yes	Air	91	Yes	No
30	Yes	No	No	No	Yes	SF_6_	105	Yes	Yes
31	Yes	No	No	No	Yes	SF_6_	73	Yes	No
32	Yes	No	No	No	Yes	PFO/ SF_6_	136	No	
33	Yes	No	No	No	Yes	SF_6_	78	Yes	No

RD; retinal detachment, SO; silicone oil

After performing this analysis, it was clear that the majority of poor outcomes defined as bleeding at either the sclerotomy or retinotomy site and intraoperative retinal detachment occurred after making the sclerotomy and before placing the fibrin implant, suggesting it was due to the excessive time needed to address choroidal bleeding after a full-penetration sclerotomy. As such, a modified surgery was proposed to limit the time of scleral opening by precauterizing the choroid and prevent bleeding. The modified procedure replaced the full thickness sclerotomy incision with a three-step sclerotomy: (1) dissection of the sclera to expose the choroid, (2) cauterization of the choroid, and (3) incision of the choroid to complete the sclerotomy (Fig. 2).

The comparison of surgical outcomes with or without the use of choroidal cautery is summarized in Table 2. Of a total of 10 cases, only 1 unintended retinal detachment (10%) was observed and 1 exhibited bleeding from the choroid (10%); no bleeding from the retinotomy (0%) was observed. Of 10 cases, 9 resulted in successful implantation (90%), with 8 exhibiting no other complications resulting in survival to term (80%). Statistically, both the incidence of unintended retinal detachment (*P* = 0.03) and bleeding from the retinotomy (*P* = 0.005) were significantly different between the standard and modified surgery group. However, bleeding from the sclerotomy (*P* = 0.4), successful implantation (*P* = 0.3), and survival to term (*P* = 0.2) between the two groups did not reach statistical significance.

**Table 2.** Summary of Surgical Outcomes

	No. of Cases	Retinal Detachment	Bleeding From Retinotomy	Bleeding From Sclerotomy	Successful Implantation	Total Time of Transplant (s)	Survival to Term^a^
Choroidal cautery –	23	11	9	5	17	568±327	13
Choroidal cautery +	10	1	0	1	9	121±41	8
*P* value		0.03^*^	0.005^*^	0.4	0.3	0.0004^*^	0.2

Table reports the values of various surgical outcomes in the general procedure (choroidal cautery -) and the modified procedure (choroidal cautery +). Total times of transplant from incision are shown with mean and standard deviation.

a Indicates animals not sacrificed during or shortly after surgery owing to complications observed during surgery.

* *P* < 0.05; χ^2^ test or one-way analysis of variance.

**Figure 4.**
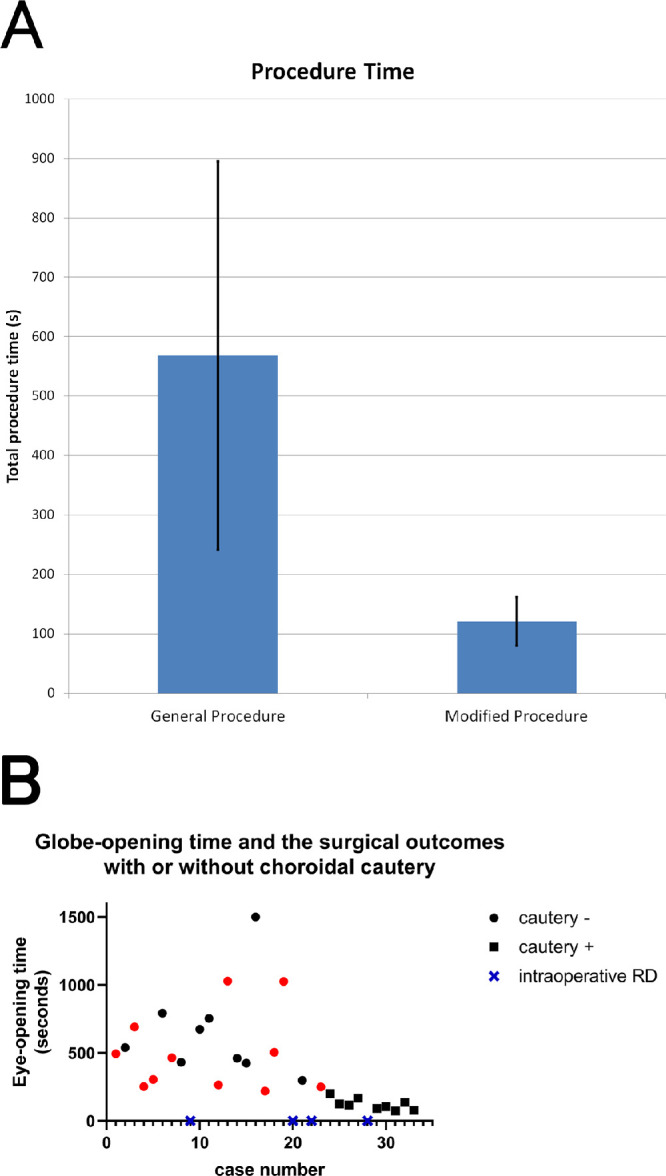
A comparison of the implantation procedure time between general and modified procedure. (A) The implantation procedure time was measured from the full-thickness sclerotomy to the first suture closure after the fibrin hydrogel deployment. The graph showed the modified procedure significantly reduced the implantation procedure time from 568 ± 327 to 121 ± 41 seconds (*P* < 0.001, one-way analysis of variance). (B) Globe-opening time and the surgical outcomes with or without choroidal cautery. Each plot of time spent per surgery and/or complications against case number was represented in the graph. The *black boxes* and *circles* were represented as successful hydrogel deployment with or without the use of cautery, respectively. The *red circles* indicate bleeding at either the sclerotomy or retinotomy site, and the *blue crosses* indicate intraoperative retinal detachment.

The total time for transplantation was also calculated for both groups. This factor was defined as the time from the full-thickness scleral and choroid incision to the time of placing the first suture, which was composed of the management of bleeding from the sclerotomy site and hydrogel deployment. This window was defined to best measure the duration of instability within the eye, owing to the fluctuating IOP and possible convective BSS outflow. The average total time of transplantation for the standard surgery group (no cautery; *n* = 23) was 568 ± 327 seconds and 121 ± 41 seconds for the modified surgery group (cautery; *n* = 10). This difference was statistically significant (*P* = 0.0004) (Fig. 4A). Taking the surgeon's learning curve into account, each plot of time spent per surgery and/or complications against case number was represented in Figure 4B, showing a deviation of the globe-opening time along with fewer complications when the choroidal cautery was applied, whereas the globe-opening time fluctuated even after surgeries were repeated without cautery.

In a subset of surviving pigs, retinal attachment was achieved in 2 of 5 (40%) without tamponade, 7 of 10 (70%) with air, 2 of 3 (67%) with silicone oil, and 2 of 3 (67%) with SF_6_ tamponade. PFOs were used selected number of cases (case 25, 27, and 32) for better visualization of the transplanted hydrogel and/or the iPSC–RPE and followed by the fluid–air exchange. Cases 25 and 27 showed no postoperative retinal detachment and case 32 underwent successful implantation but was nonsurvival.

### Disposable Tip Design and Prototypes

The analysis revealed a significant outflow of BSS from the sclerotomy, even after the first-generation device was inserted into the eye. Initially, the first-generation inserter was designed with a stainless steel tube owing to ease of prototyping, the mechanical strength of the device, and the ability to articulate the device within the eye for precision implantation.

**Figure 5.**
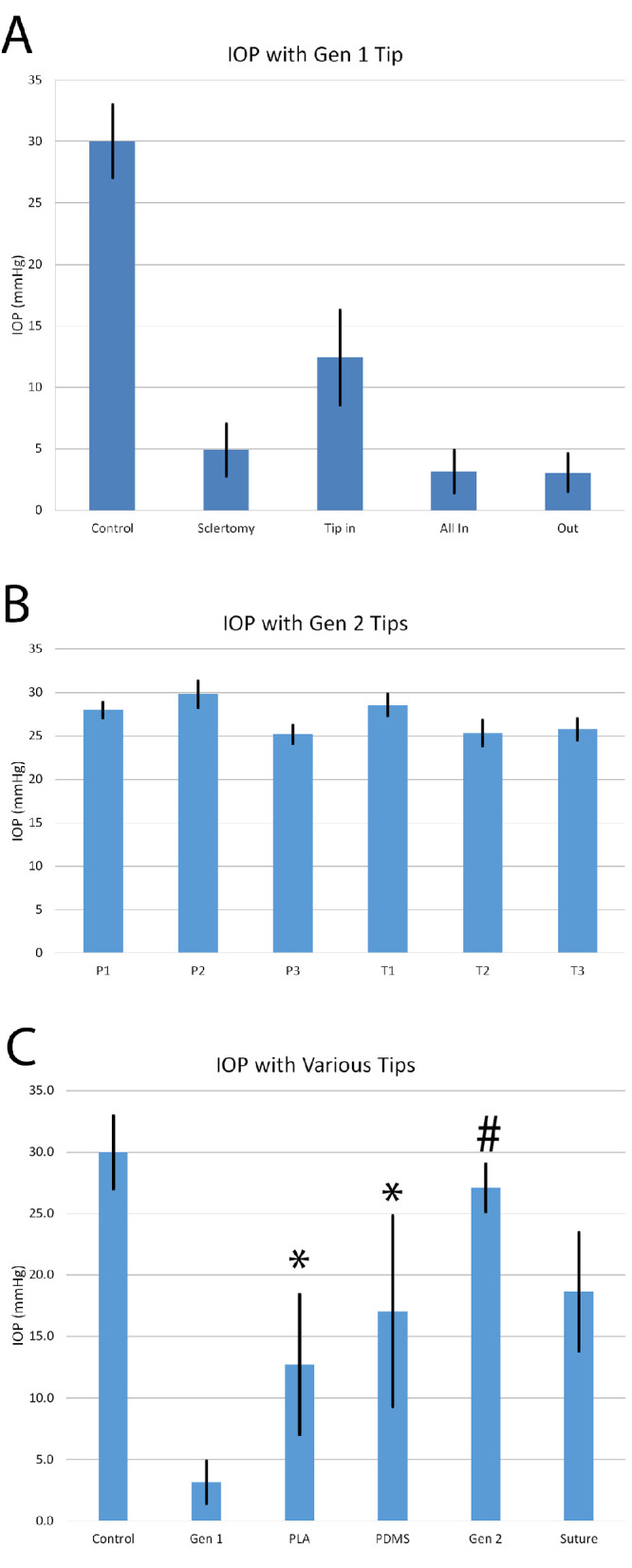
Ex vivo IOP measurements across various surgical device designs. (A) Graph of measured IOP at various stages of the general procedure, demonstrating reduced IOP throughout the procedure using the first generation surgical tip at various depths. (B) Graph of measured IOP using multiple prototypes of the second-generation surgical tip. The range of measured IOP between all devices was 25.2 to 29.8 mm Hg. (C) Graph of measured IOP across the various devices, resulting in statistical differences (*P* < 0.001). Between groups, the PLA (*P* = 0.004) and PDMA plugs (*P* < 0.001) significantly increased IOP compared with the no attachment first generation tip. The second-generation tip (*P* < 0.001, Tukey's HSD test) maintained IOP which was statistically significant from all other groups except control (no incision).

To quantify the instable IOP of the eye during the transplantation, we performed ex vivo experiments to measure the IOP during surgery. The IOP was maintained in the eye during the procedure by the gravity pole setup of the Accurus instrument set at 30 mm Hg throughout all the experiments. The average measured IOP before an incision was 30.2 ± 3.0 mm Hg (control). Upon making the 3.6-mm scleral incision using a slit knife, the measured IOP decreased to 5.0 ± 2.1 mm Hg (Fig. 5A). When the first-generation tip was initially placed into the sclerotomy (Fig. 5A) tip-in phase, the clear rectangular housing filled the bulk of the sclerotomy and the IOP was measured at 12.5 ± 3.9 mm Hg. Once the inserter was properly aligned with the site of implantation (the all-in phase), the IOP decreased to 3.2 ± 1.8 mm Hg. The IOP remained the same when the inserter was completely removed (3.1 ± 1.5 mm Hg). At the end of the experiment, a single 8-0 Vicryl suture was placed in the middle of the incision and the IOP was measured at 18.7 ± 4.9 mm Hg. These data indicated that the IOP was not maintained during implantation and remained unstable.

Because it was suspected that the bulk volume of the clear plastic tip helped to maintain some pressure, two-plug–style attachments were prototyped to dock with the first-generation inserter hub (Supplementary Fig. S1). These devices dock with the sclerotomy, whereas the first-generation tip is aligned with the site of implantation. Both the IOP stabilizer devices maintained an increased IOP with mean measured values of 12.7 ± 5.7 mm Hg (PLA plug) and 17.1 ± 7.8 mm Hg (PDMS plug), similar to the pressure measured during the tip-in phase with the first-generation tip. Although these prototypes improved the maintenance of the IOP, the outflow force coming from the sclerotomy made it difficult to position and set the plug in the appropriate orientation and maintain it while articulating the implanter.

To take advantage of the increase in IOP afforded by the stabilizer devices, a second-generation disposable inserter tip was developed (Fig. 3A) that maintains the same external geometry across the full hub to ensure filling the sclerotomy and maintaining IOP (Fig. 3B). The tip was designed to be compatible with the previous actuator handle. The inner geometry was similar to the first generation device, providing sufficient clearance to fit a 1.5 mm × 5.0 mm × 200 µm implant. The clearance allowed loading of a fibrin-scaffolded iPSC–RPE implant without scrapping off the cell monolayer (Fig. 3C). The outer dimensions of this second-generation tip are 1.29 mm tall by 2.36 mm wide (Fig. 3D). To enable proper orientation of the tip to the retinotomy site without putting excess strain on the sclera, we prototyped various radii of curvature (P1–3) and angle of bevel (T1–3). For proper alignment of the tip to the site of implantation in a pig eye, a radius of curvature of 40 mm and a bevel of 10° was selected per the surgeon's experience.

The ex vivo IOP experiment was repeated with three tips from two separate batches of the second-generation tip. The mean IOPs for the individual devices, when aligned to the site of implantation, were 28.0 ± 0.9 mm Hg (P1), 29.8 ± 1.5 mm Hg (P2), 25.2 ± 1.1 mm Hg (P3), 28.6 ± 1.2 mm Hg (T1), 25.3 ± 1.5 mm Hg (T2), and 25.8 ± 1.2 (T3) (*n* = 10), suggesting a very low variability between devices (Fig. 5B). As a group, the average of the second-generation tip was 27.1 ± 1.9 mm Hg, which was significantly higher than the all other groups (*P* < 0.001, Tukey's honest significant difference test) (Fig. 5C). Furthermore, three separate surgeons reported the ease of insertion and manipulation of the device.

### iPSC–RPE + Fibrin Implantation

To apply the lessons learned from the improved surgical technique and second-generation inserter tip, we set out to quantify outcomes in four nonsurvival pig surgeries using fibrin-scaffolded iPSC–RPE implants. These implants were generated as previously described^12^ and loaded into the second-generation tips for use.

**Figure 6.**
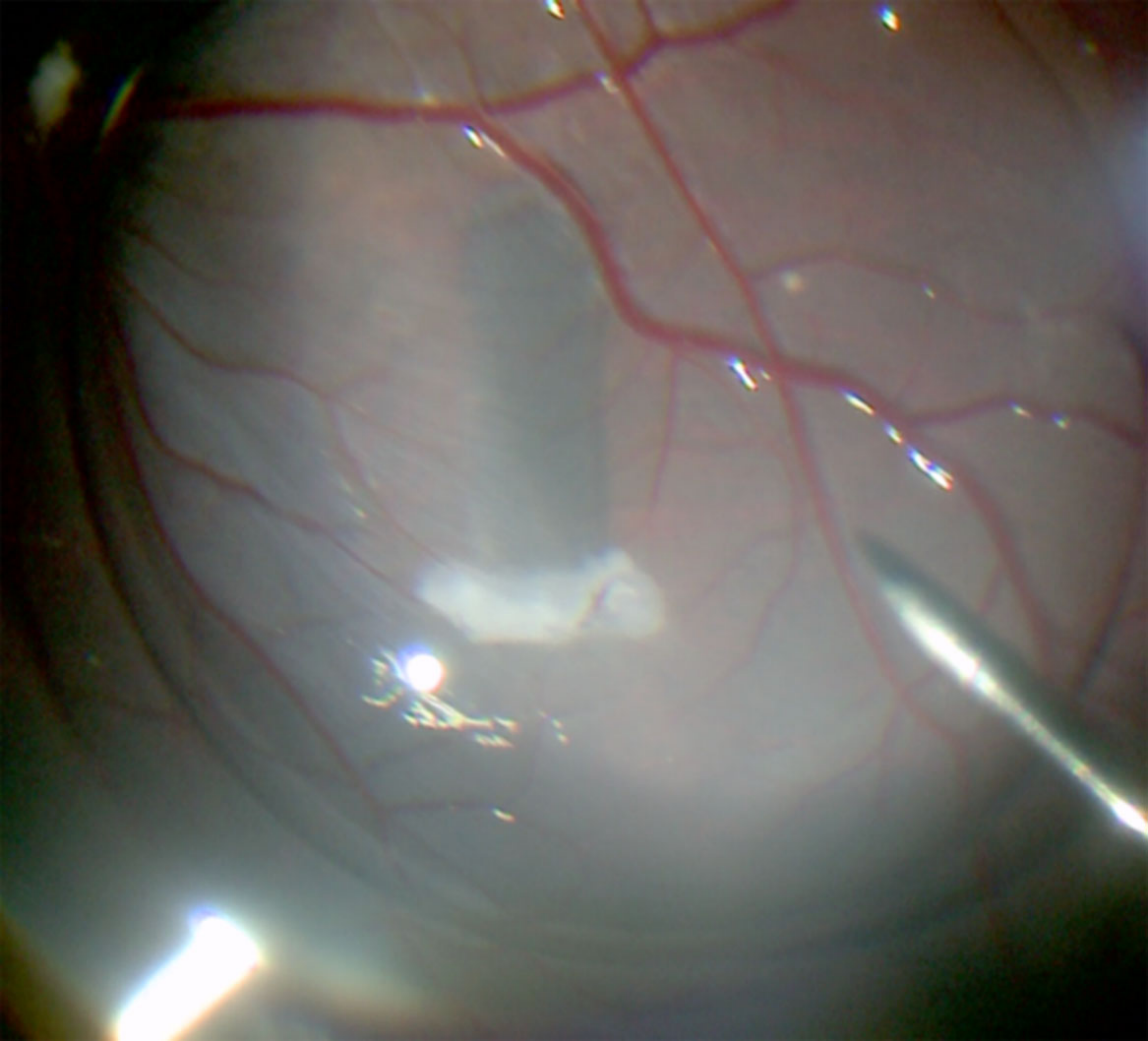
Implantation of MRPE611. Photograph demonstrating successful placement of MRPE611 into the subretinal space of a pig eye. PFO was used to reattach the retina for improved visualization of MRPE611.

Out of a total of four cases, no cases of unintended retinal detachment, one case of bleeding from retinotomy, and no cases of bleeding from choroid were observed (Table 3). Implantation was successful in all four cases. The total time of transplantation was 153 ± 30 seconds. Upon transplantation and flattening of the overlying retina, the RPE-based implant was visible in the subretinal space (Fig. 6). Qualitatively, there did not seem to be any scraping of the RPE monolayer in any of the four cases.

**Table 3.** Summary of Surgical Outcomes With MRPE611 Implantation Using Modified Procedure and Second-generation Tips

	No. of Cases	Retinal Detachment	Bleeding From Retinotomy	Bleeding From Sclerotomy	Successful Implantation	Total Time of Transplant (s)
Cautery + V2 tip + iPSC-RPE implant	4	0	1	0	4	153 ± 30

Table reports the values of various surgical outcomes in MRPE611 implantation. Total times of transplant from incision are shown with mean and standard deviation.

## Discussion

During the development of our surgical technique, we noted a hazy view during hydrogel deployment, which we later attributed to the decreased IOP replicated in our ex vivo experiment. We also hypothesized that the major complications initially encountered (choroidal bleeding, retinal detachment, retinotomy bleeding) resulted from the long time required to control choroidal bleeding after the scleral incision and an extended period of ocular hypotony. The choroidal bleeding was an unexpected complication; typically, pars plana incisions in humans are not associated with excessive bleeding. This factor seems to be an anatomical difference between humans and pigs.^16^ Although our observation for the depth of pars plana in a cadaver pig eyes revealed that the ora serrata was 3.5 mm from the limbus, the variability in the depth was far too great to be predictably accurate during live surgery. Apparently, some pig species seem to have additional retinal vasculature in the nasal region close to the ora serrata, which could explain the excessive bleeding from the sclerotomy site. Another possible reason for this bleeding might be from the circumferential choroidal vasculature fed by a long posterior ciliary artery that was frequently observed while making the sclerotomy.^16^ Regardless, precauterization of the choroid helped to decrease the incidence of choroidal hemorrhage, decreasing the total time of the implantation. Preplaced scleral sutures also allowed a surgeon to close the wound immediately after the implantation. Thus, the modified transplant procedures significantly decreased the incidence of complications during surgery and increased the success rate of the implantation surgery.

In studies that stretch back to the 1990s, it has been shown that transplantation of RPE monolayers provides superior outcomes over subretinal injection of a cell suspension, with dramatically improved successful engraftment of transplanted cells.^2^^,^^4^^,^^5^ However, with this practice, the complexity of the surgery is increased owing to the technical need to insert a larger instrument that can accommodate the geometry of a monolayer and potentially an associated scaffold. Some advocate using 20 G^17^^–^^19^ or 17 G device^7^; other investigators altered an intraocular lens inserter.^20^^,^^21^ Those instruments are still considered to be a large devices in the era of microincision vitrectomy surgery. For example, Kamao et al.^17^ used a fluid flow path with a 20 G catheter to guide the RPE monolayer into the subretinal space. Because they used fluid flow, the surgery required two retinal incisions, namely, an inlet and outlet. The graft folding and misdirection were detected in some cases postoperatively, probably because they did not use a scaffold during the deployment, and the positioning of the floating RPE monolayer was complicated by the flow path.^17^ Stanzel et al.^18^ tested a 20 G metallic shooter instrument and proposed gelatin encapsulation of an RPE monolayer seeded on polyester implants. Gelatin-coated implants were ejected smoothly in 12 of 14 surgeries (86%), whereas naked implants frequently became trapped within the instrument, which decreased the success rate to 53%. From these examples, it is essential to note that the transferring device must be designed with the specific scaffold in mind. Fernandes et al.^7^ invented a retractable injector that can curl a thin synthetic parylene substrate (3.5 × 6 mm) to decrease the incision size to 1.5 mm. Although curling the implant does decrease the incisional size requirement, it also increases the risk of cell loss during implantation. For example, a clinical trial using this device seems to show a significant zone around the edge of the implant with missing RPE.

In our initial design assessment, we settled on a few key features of the device: a mechanical plunger, clear implant housing, compatibility with current vitrectomy systems, and a curved shaft. We decided on a mechanical plunger in place of a fluid convective deployment^17^ for several reasons. First, mechanical deployment increases the efficiency and control of implant deployment. Second, the use of fluid convective deployment would require either greater fluid flow for deployment or the inclusion of a greater viscosity fluid. The concerns arising from fluid convective flow included incidental damage to the subretinal architecture, a requirement for inlet and outlet for the retinal bleb, or the need to use a higher viscosity fluid. Use of a higher viscosity fluid, like hyaluronic acid, has a greater potential to shear the transplant cells and is known to prolong retinal detachments,^22^ potentially complicating RPE integration and degradation of a degradable scaffold. The clear implant housing was designed to allow the surgeon to inspect the implant before placement, as well as verify the polarity of placement, which is further ensured by the curvature of the instrument tip. We also wished to minimize errors owing to hand stabilization during deployment; thus, we settled on a pneumatically driven actuator that is compatible with the current vitrectomy system and a foot pedal.

A great deal of attention has recently been given to the development of suitable scaffolds for RPE transplantation; the development of surgical procedures with emphasis on IOP management during implantation has lagged.^17^^,^^23^^,^^24^ We have tested various types of IOP stabilizers that were designed to prevent outflow from the sclerotomy site during the hydrogel deployment. As we expected, both designs tested were able to maintain the IOP significantly higher than the standard injector device. Interestingly, we saw no difference between the two types of plugs, both of which maintain some pressure. However, the outflow coming from the sclerotomy made it difficult to position and set the plug in the appropriate orientation and maintain it while articulating the implanter. The rigid PLA plug allowed for better maneuverability than the soft PDMS plug during hydrogel deployment. This factor led us to redesign the surgical tip to include a single external dimension across the shaft. Using an extrusion process also helped to maintain a tight tolerance and a more oval shape that better fits the linear scleral incision compared with the more rectangular outer edge of the clear housing in the first-generation tip.

To maintain a clear view during the transplantation, it was necessary to maintain the IOP. Because the implantation requires alignment of the tip very close to the retina, maintenance of the pressure helps to shape the eye preventing the inserter from touching the retina and causing unnecessary bleeding.

Regarding the prevention of complications, multiple insertions of the device should be avoided. Multiple insertions of the device induced vitreous herniation, leading to retinal incarceration in the wound, especially under the situation of high infusion flow. Data were not quantified, yet we strongly recommend removing the vitreous around the scleral incision site aggressively, as other literature has suggested previously.^25^ In the case of misdirection of the implant or trapped by an inserter device, a recovery technique is also necessary for future clinical use. If a synthetic substrate is misoriented, it will be difficult to remove from the subretinal space.^4^^,^^5^ A benefit of using the fibrin hydrogel as a scaffold is that it can be easily broken by a vitrectomy cutter with a piecemeal approach if it is misplaced.

This study does not take into account the learning curve of the transplant surgery for the comparison of the two series of surgeries. However, we do not believe this factor alone addresses the major discrepancy between the two surgical models. Our IACUC protocol allowed for only fibrin hydrogel and/or iPSC-derived RPE transplant surgery, but not simply for in vivo IOP measurement. We have discussed the development of a reliable, reproducible, and safe subretinal RPE transplant surgery as well as the necessity to obtain a refined delivery instrument for this investigational therapy.

## Conclusions

We report the implementation of a modified sclerotomy in pig surgery to include precauterization of the choroid and a preplaced scleral suture to minimize the complications associated with a prolonged sclerotomy opening and consequential IOP fluctuations. We also report iterative design of an inserter tip to support management of IOP during the implantation.

## Supplementary Material

Supplement 1

Supplement 2
