# Supplementary material for: Methodological Approach to Improve Surgical Outcomes of a Pig Subretinal Implantation Model
Source: Transl Vis Sci Technol. 2022 Apr 29;11(4):24. doi: 10.1167/tvst.11.4.24 (PMC9055557; doi:10.1167/tvst.11.4.24)
Supplement: Supplement 1 [file tvst-11-4-24_s001.pdf]

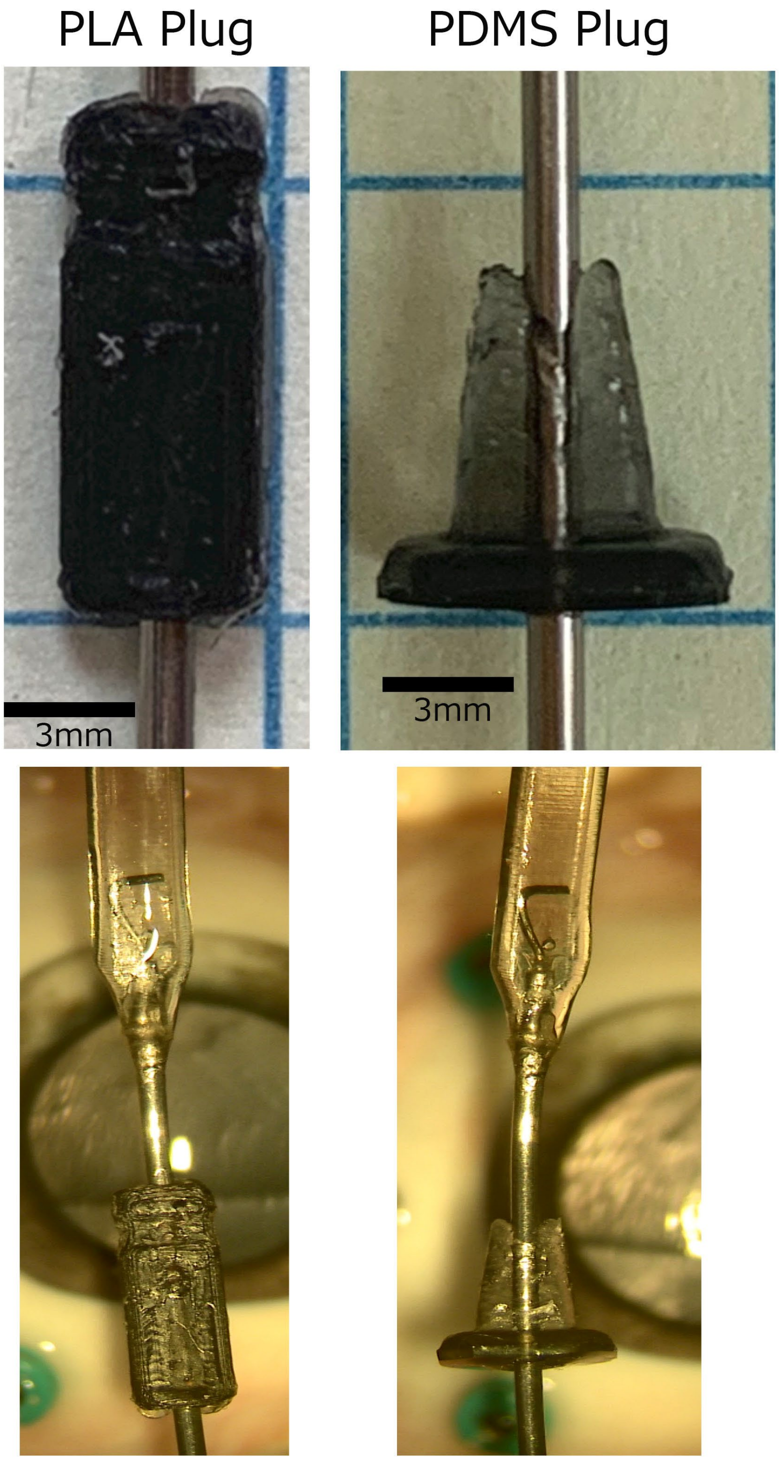

Supplemental Figure 1: Plug attachments for first generation surgical tip. Photographs depicting the PLA and PDMS plugs (top row), and the respective plugs attached to the first generation surgical tip (bottom row).
