# Supplementary material for: Methodological Approach to Improve Surgical Outcomes of a Pig Subretinal Implantation Model
Source: Transl Vis Sci Technol. 2022 Apr 29;11(4):24. doi: 10.1167/tvst.11.4.24 (PMC9055557; doi:10.1167/tvst.11.4.24)
Supplement: Supplement 2 [file tvst-11-4-24_s002.pdf]

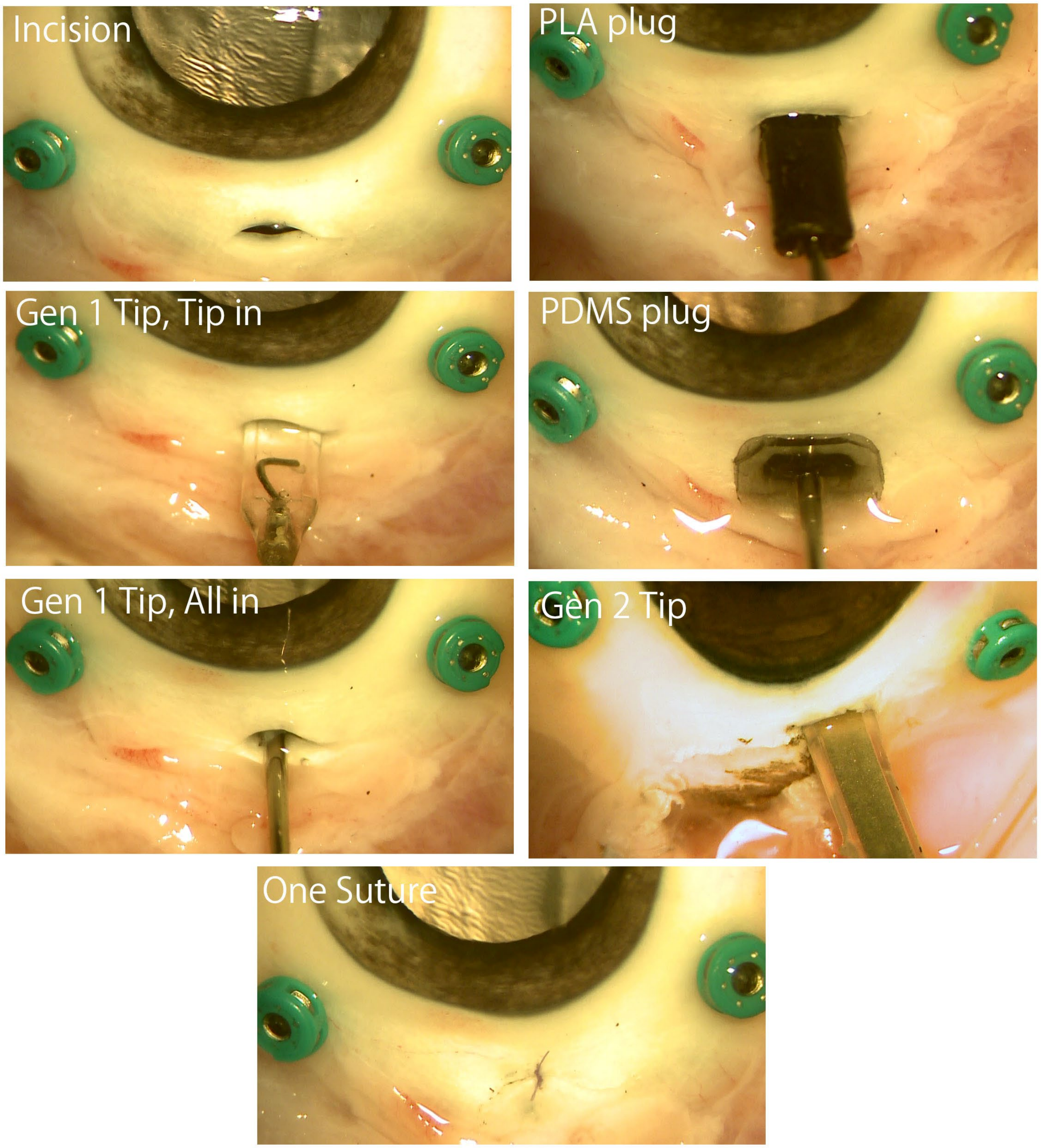

Supplemental Figure 2: Ex vivo IOP measurements with various surgical devices. Photographs representing the various conditions investigated within the ex vivo IOP measurement experiment as reported in figure 5.
